# Acquisition of Neural Action Potentials Using Rapid Multiplexing Directly at the Electrodes

**DOI:** 10.3390/mi9100477

**Published:** 2018-09-20

**Authors:** Mohit Sharma, Avery Tye Gardner, Hunter J. Strathman, David J. Warren, Jason Silver, Ross M. Walker

**Affiliations:** 1 Department of Electrical and Computer Engineering, University of Utah, Salt Lake City, UT 84112, USA; tye.gardner@utah.edu (A.T.G.); jason.silver@utah.edu (J.S.); 2 Department of Biomedical Engineering, University of Utah, Salt Lake City, UT 84112, USA; h.strathman@utah.edu (H.J.S.); david.warren@utah.edu (D.J.W.)

**Keywords:** neural recording, neural amplifier, microelectrode array, intracortical, sensor interface, windowed integration sampling, mixed-signal feedback, multiplexing

## Abstract

Neural recording systems that interface with implanted microelectrodes are used extensively in experimental neuroscience and neural engineering research. Interface electronics that are needed to amplify, filter, and digitize signals from multichannel electrode arrays are a critical bottleneck to scaling such systems. This paper presents the design and testing of an electronic architecture for intracortical neural recording that drastically reduces the size per channel by rapidly multiplexing many electrodes to a single circuit. The architecture utilizes mixed-signal feedback to cancel electrode offsets, windowed integration sampling to reduce aliased high-frequency noise, and a successive approximation analog-to-digital converter with small capacitance and asynchronous control. Results are presented from a 180 nm CMOS integrated circuit prototype verified using in vivo experiments with a tungsten microwire array implanted in rodent cortex. The integrated circuit prototype achieves <0.004 mm^2^ area per channel, 7 µW power dissipation per channel, 5.6 µV_rms_ input referred noise, 50 dB common mode rejection ratio, and generates 9-bit samples at 30 kHz per channel by multiplexing at 600 kHz. General considerations are discussed for rapid time domain multiplexing of high-impedance microelectrodes. Overall, this work describes a promising path forward for scaling neural recording systems to numbers of electrodes that are orders of magnitude larger.

## 1. Introduction

Penetrating microelectrodes that record neural signals currently achieve the highest temporal and spatial resolution available for measuring nervous system activity in vivo [1,2]. Multichannel electrode arrays can be implanted into regions of the central and peripheral nervous systems, and extracellular measurements of neuronal activity can be taken by interfacing the electrodes to signal acquisition electronics that are composed of amplifiers, filters, and analog-to-digital converters (ADCs). This general approach is frequently used in experimental neuroscience [3,4,5], as well as in research toward prosthetics and brain-computer interfaces that are directly controlled by neural activity [6,7,8]. Recently, neural interface technologies based on microelectrode arrays have seen dramatic growth and interest that has been supported by progress in materials, fabrication, electronics, and neuroscience [9].

A key goal for the future is to scale the number of implanted electrode sites up by orders of magnitude. In neuroscience, this goal is important for accessing larger populations of neurons to provide a more complete view of information processing in the brain. For medical applications of neural interfaces, scaling has great potential to provide more effective therapies and prosthetic devices. Regions of the human neocortex contain approximately 50,000 neurons/mm^3^ [10], but neural interfaces used in human clinical trials [6,7,8] can only provide cellular-level signals from around 200 different neurons sampled in a roughly 10 mm^3^ volume of tissue. Today, most microelectrode arrays have between 16 and 256 electrode sites, typically positioned at a few hundred micrometer spacing (<10 sites/mm^2^) [1,2]. There are large ongoing efforts focused on scaling up to thousands of electrodes and beyond [11,12,13,14,15]. These projects often target orders of magnitude higher density of the electrode sites (>1000 sites/mm^2^) [13,14,15]. Fully implantable acquisition electronics are needed to support this scaling, because passive wiring of each electrode site to external electronics incurs a number of surgical and reliability problems [1,16].

**Figure 1:**
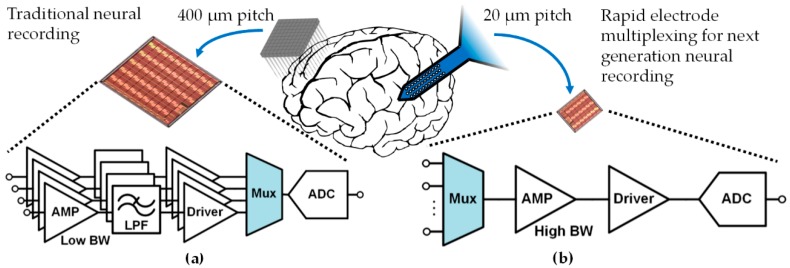
(**a**) Conventional neural recording electronics use individual amplifiers and filters dedicated to each electrode, and often employ back-end multiplexing to a smaller number of analog-to-digital converters. (**b**) Rapid multiplexing directly at the electrodes can be used to share amplifiers and filters across many electrodes, leading to a drastic reduction in the size of the electronics. Small channel area enables high-density arrays with active electronics closer to the electrode.

Massive system scaling requires reconsideration of the traditional approach to designing acquisition electronics. The conventional approach is to use individual amplifiers and filters dedicated to each electrode site (Figure 1a). While this approach offers simple solutions to many of the technical issues involved in acquiring neural signals, the resulting silicon chip area per channel is too large to support thousands of electrodes and beyond without dominating the size and form factor of a fully implantable microsystem. Progress has been made in reducing the size of traditional acquisition electronics (e.g., there are >2000 articles listed under “neural amplifier” in the Inspec database at the time of this writing), but state-of-the-art designs still typically result in 0.04–0.1 mm^2^ of chip area per electrode channel (10–25 channels/mm^2^) [17].

A promising approach for reducing the area of the acquisition electronics is to use time division multiplexing to rapidly sample multiple electrode sites with a single front-end circuit, without preamplification (Figure 1b). However, rapid multiplexing directly at the electrodes raises challenges related to electrode offsets and aliasing of high-frequency noise. These challenges have been shown to be tractable for non-penetrating microelectrode arrays used in electrocorticography (ECoG) [18,19], but these low-impedance arrays measure average neural activity from large groups of neurons and generally do not provide the spatial or temporal resolution needed to observe action potentials.

To the authors’ best knowledge, this paper presents the first in vivo demonstration of a rapidly multiplexed neural recording system without preamplification, designed for high-impedance, penetrating microelectrode arrays that provide measurements of action potentials (APs). Time division multiplexing is often used after amplification to reduce ADC area [17,20,21,22,23] (Figure 1a), and has also been used for serialized analog communication in neural recording systems [24,25,26]. However, these prior works all use multiplexing after amplification, which does not reduce the area of the amplifiers and thus has limited benefit. In the work presented in this report, a 180 nm CMOS circuit was designed to address issues from electrode offsets and high-frequency noise aliasing that become problematic when rapidly multiplexing electrodes directly to a single front-end amplifier, without preamplification, in order to enable drastic area reductions.

The rest of this paper is organized as follows. Section 2 describes the fundamental theory of acquiring rapidly multiplexed signals from microelectrode arrays without preamplification, and presents informative electrode characterization measurements. Section 3 proposes a circuit architecture for rapid multiplexing directly at the electrodes, along with details of a fabricated prototype. Section 4 presents test results for the prototype, including in vivo experiments with signals recorded from the rodent cortex. Section 5 provides a discussion and implications for future research.

## 2. Rapidly Multiplexed Neural Recording: Theory and Practical Issues

**Figure 2:**
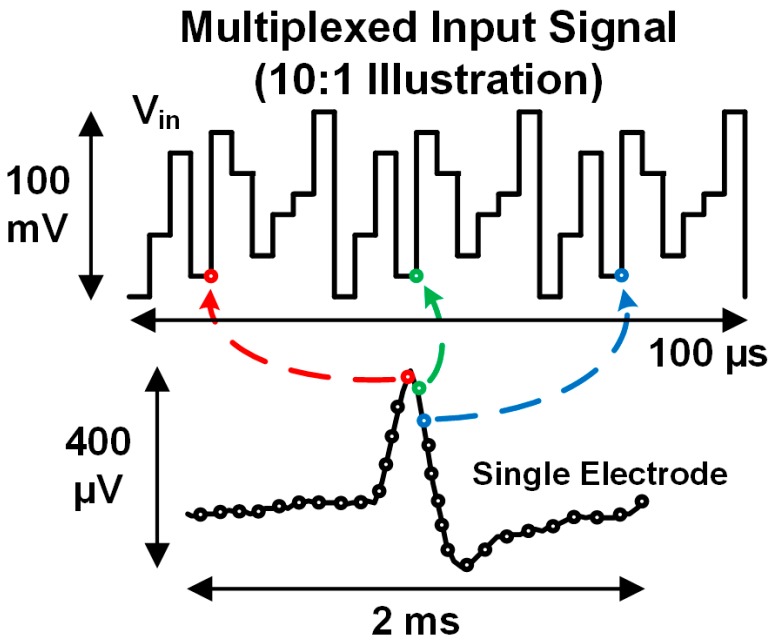
Illustration of the signal that arises from rapidly multiplexing multiple electrodes to the input of a single acquisition circuit. The amplitude of the signal is dominated by DC offsets from differences in polarization potentials between recording and reference electrodes. The goal is to acquire much smaller action potential signals, which are multiplexed in the time domain.

Figure 2 illustrates the input signal that arises from rapidly multiplexing multiple electrode sites to a single front-end amplifier. This paradigm uses time domain multiplexing to switch between electrodes at a high enough rate (e.g., 600 kHz) such that each different electrode can be sampled at a frequency that is typically used when acquiring APs (e.g., $f_{ch}=$ 30 kHz). Since each electrode is visited for only a short time (microseconds or less) within the overall sampling period, the total electrode voltage appears as a step input to the electronics, which confounds traditional filtering. As a result, the multiplexed signal amplitude is dominated by DC offsets arising from polarization potentials at the interfaces between electrodes and the tissue [27]. To accurately sample the smaller AP signals, the bandwidth of the recording electronics must be made large such that the step transients can settle fully. This large bandwidth creates challenges for achieving adequate signal-to-noise ratio (SNR) in the presence of high-frequency noise. Overall, rapid multiplexing without preamplification requires more careful consideration of electrode properties than the traditional approach. This section describes the fundamental challenges to rapid multiplexing, and offers high-level solutions.

### 2.1. DC Offsets from the Electrodes

Electrochemical polarization naturally develops an equilibrium potential at the electrode‒tissue interface [27]. Commonly, a single low impedance reference electrode is used when recording from a multichannel electrode array, e.g., a single platinum wire that is separate from the array itself [27]. The reference electrode develops its own equilibrium potential, which differs significantly from the equilibrium potentials of the recording electrodes. The difference between the potentials of a recording and reference electrode manifests as a DC offset signal when recording. These DC offset voltages are typically much larger than APs, and can be in the 1–50 mV range depending on the materials and geometries of the recording and reference electrodes (when performing “bipolar” recording between two identical electrodes, the DC offsets are usually smaller but still often within the millivolt range). Unfortunately, quantitative studies of DC offsets seen during recording are generally lacking in the literature. Differences also exist between the equilibrium potentials of each recording electrode in a multichannel array, due to manufacturing tolerances that result in physical differences, as well as differences in the local chemistry around each implanted electrode [27]. The DC offsets observed during recording also depend on the input impedance of the acquisition electronics and any leakage currents from protection diodes or other devices [28].

Dedicating an individual amplifier to each electrode site (Figure 1a) [17,20,21,23,29,30,31] allows simple high-pass filtering to separate the electrode offsets from APs before amplification up to the full scale range of the ADC (~1 V). When rapidly multiplexing before amplification, a high-pass filter is not feasible, because the DC offsets and APs both appear as a step input and do not change appreciably during the time allotted for generating a sample (microseconds or less). Indeed, applying such a high-pass filter to the waveform illustrated in Figure 2 would simply remove its average value.

One solution is to limit the overall gain in the signal path and use a high-resolution ADC to digitize both the modulated offset signal as well as the much smaller APs. However, assuming that the DC offsets are 50× larger than the APs, this approach requires an additional 6 bits of resolution (~16 bits in total). While such resolution is achievable, the resulting ADC specifications are challenging to achieve with low power and low circuit area, since the ADC must also run at a high sample rate of $f_{s}=M\cdot f_{ch}$, where *M* is the number of multiplexed electrodes. The architecture presented in this report uses a mixed-signal DC offset rejection approach that greatly reduces the burden on the ADC by avoiding the need for increased resolution.

### 2.2. Noise from Acquisition Electronics

Whether employing rapid multiplexing (Figure 1b) or the traditional approach (Figure 1a), noise from the acquisition electronics must be made low enough to accurately acquire AP signals. The target noise specification depends on the intrinsic signal quality that can be achieved with particular microelectrodes. There is a great incentive to avoid overdesigning the electronics in terms of noise performance, because circuit power dissipation trades directly with thermal noise power [32]. The most common philosophy when targeting fully implantable electronics is to roughly match the circuit noise specification to the background noise level from the electrodes (5–10 µV_rms_, as discussed in Section 2.3), so that there is a significant, yet non-dominating, contribution of noise from the electronics. In a traditional neural recording circuit design, the bandwidth of the signal path is generally chosen in the 5–10 kHz range [17], stemming from the spectral content of APs (0.5–5 kHz) [33]. This low bandwidth limits noise, and hence allows reduction of power dissipation.

Noise equivalent bandwidth (NEB) is a useful metric for comparing the traditional and rapidly multiplexed approaches to neural recording. The NEB of a filter response, H(f), is defined as the bandwidth of a brick-wall filter that would pass the same amount of noise:

(1) $$NEB≜\int_{0}^{\infty }{\left|H\left(f\right)\right|}^{2}\;df.$$

For example, a single pole response has a NEB equal to $\pi f_{p}/2$, where $f_{p}$ is the pole frequency.

To acquire rapidly multiplexed APs, the bandwidth of the acquisition channel must be large enough to allow complete transient settling of the multiplexed waveform (Figure 2) in the time period allocated to each electrode (microseconds or less). The settling requirement necessitates a larger bandwidth than the traditional approach. Assuming instantaneous voltage sampling with a single pole low-pass filter response, transient settling necessitates a bandwidth of

(2) $$f_{BW}=\frac{-\ln\left(\varepsilon_{dtol}\right)}{2\pi T_{s}},$$

where $\varepsilon_{dtol}$ is the tolerable dynamic settling error (0.1% for 10-bit accuracy), and $T_{s}$ is the amount of time available for settling. To achieve a per-channel sampling rate of $f_{ch}$ (~30 kHz) across M multiplexed electrodes, the ADC must sample at a rate of $f_{s}=M\cdot f_{ch}$, allowing a maximum settling time of $T_{s}=1/f_{s}$ for each electrode. The NEB for multiplexed acquisition using instantaneous voltage sampling thus increases proportionally to M, in order to maintain $f_{ch}$ for each electrode:

(3) $$NEB_{vs}=\frac{\pi }{2}f_{BW}=-f_{s}\ln\left(\varepsilon_{dtol}\right)/4=-Mf_{ch}\ln\left(\varepsilon_{dtol}\right)/4.$$

This increased *NEB* necessitates a reduction in the acquisition circuit’s noise power spectral density (PSD) to maintain the same total integrated noise specification as a traditional neural recording circuit, since noise at frequencies higher than $f_{ch}/2$ will alias and show up in the samples. Reducing the wideband noise PSD is achieved by investing more power in the amplifier (Amp in Figure 1b) to reduce its input-referred thermal noise. Assuming that $f_{ch}$ is chosen to be 3× the AP bandwidth (a typical case), the NEB of a multiplexed acquisition circuit (Figure 1b) that uses instantaneous voltage sampling increases above the NEB of a traditional acquisition circuit (Figure 1a) according to

(4) $$NEB_{vs}=-M\ln\left(\varepsilon_{dtol}\right)\left(3/2\pi \right)NEB_{trad},$$

where $NEB_{trad}$ is the NEB of a traditional neural recording circuit (Figure 1a). Assuming $\varepsilon_{dtol}=0.1\%$ to allow accurate detection of APs in the presence of large DC offsets, $NEB_{vs}≅3.5M\cdot NEB_{trad}$. Assuming a one-to-one tradeoff between the circuit’s thermal noise PSD and its power dissipation (the typical case [32]), the NEB enlargement translates to 3.5× higher power per channel for a multiplexed acquisition circuit that uses instantaneous voltage sampling, compared to a traditional neural recording circuit with the same total integrated noise. This power penalty is quite large, but can be avoided by alternative sampling methods. In this work, windowed integration sampling (WIS) was used to reduce the NEB of the rapidly multiplexed acquisition circuit by ~3.5× versus instantaneous voltage sampling, restoring the power per channel back to the traditional range.

Windowed integration sampling (WIS) [31,34,35] is an alternative to instantaneous voltage sampling that breaks the tradeoff between settling accuracy and NEB. The fundamental idea is to integrate the signal over a finite window of time, and then sample the result. Integration reduces high-frequency noise according to a sinc characteristic in frequency, which can be understood by examining the Laplace transform of a continuous moving average function:

(5) $$\;y\left(t\right)=\int_{t-T_{s}}^{t}v_{in}\left(t\prime \right)dt\prime \;$$

(6) $$\left|𝓛\left\{y\left(t\right)\right\}\right|=\frac{\left|2\sin\left(\omega T_{s}/2\right)\right|}{\omega }\left|v_{in}\left(\omega \right)\right|,$$

where 𝓛{·} is the Laplace transform and ω is frequency. The samples $y\left(nT_{ch}\right)$ contain aliased high-frequency noise of $v_{in}\left(\omega \right)$, but the sinc magnitude response attenuates high frequencies with a NEB that depends on the duration of $T_{s}$, which is shown by analyzing Equation (6) with a normalized DC gain of 1:

(7) $$NEB_{wis}=\int_{0}^{\infty }\frac{\sin^{2}\left(\pi fT_{s}\right)}{{\left(\pi fT_{s}\right)}^{2}}df=\frac{1}{\pi T_{s}}\int_{0}^{\infty }{\mathrm{sinc}}^{2}\left(x\right)dx=\frac{1}{2T_{s}}.$$

The NEB of WIS can be written in terms of the NEB of the traditional neural recording approach as

(8) $$NEB_{wis}=M\left(3/\pi \right)NEB_{trad}.$$

Thus, the power consumption per channel of a multiplexed system using WIS can be about the same as a traditional system, while the area per channel can be divided by M. Multiplexing with voltage sampling incurs an extra multiplicative penalty of $\ln\left(\varepsilon_{dtol}\right)/2$ compared to WIS (see Equation (4)). For example, given M= 10, $f_{ch}=$ 30 kHz ($T_{s}≅$ 3.3 µs), $\varepsilon_{dtol}=$ 0.1%, the NEB of instantaneous voltage sampling is 518 kHz while the NEB of WIS is only 150 kHz, a factor of 3.5× smaller.

Besides thermal noise, MOSFET devices display 1/*f* noise (also called “flicker” or “pink” noise), which originates from trapping and de-trapping of carriers in states close to the interface between the silicon and gate insulation material [36]. This noise source is substantial in the AP band, and motivates large transistor sizes to reduce its PSD. Interestingly, the 1/*f* noise requirements and tradeoffs are the same for both traditional and rapidly multiplexed acquisition circuits, which can be seen by considering that each channel is sampled at $f_{ch}$ in both approaches, leading to a discrete time spectrum confined to $f_{ch}/2$ for both. This leads to similar transistor sizes for both approaches, although the area is amortized across channels when using rapid multiplexing.

With an equivalent power per channel that is neither worse nor better than a traditional neural recording circuit, the rapidly multiplexed approach can be seen as combining all the power consumption that would be spent in multichannel circuitry into one faster circuit that uses dynamic operation (speed) to reduce circuit area. This strategy generally leverages one of the main strengths of CMOS technology, i.e., high-speed operation, which is not utilized by the traditional approach.

### 2.3. Noise from the Electrodes

Excluding noise from the acquisition electronics, the noise floor for the traditional approach to AP recording (Figure 1a) is dominated by biological activity in the AP band [33], with a relatively minor contribution from thermal noise generated by the electrode, tissue, and their electrochemical interface. When rapidly multiplexing, however, this thermal noise becomes an increasing concern due to the larger bandwidth required. High-frequency noise from the electrode‒tissue system will alias into the AP spectrum, and increase the variance of the acquired samples. WIS is effective in reducing the contribution of high-frequency noise, but residual aliased noise still presents a limit on the number of electrodes that can be multiplexed while achieving an acceptable noise floor and SNR.

**Figure 3:**
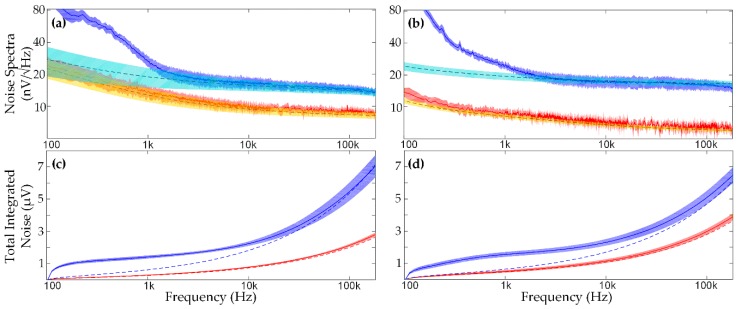
Noise characterizations for representative 16-channel microelectrode arrays. (**a**) Silicon microelectrodes (Blackrock Microsystems) and (**b**) microwire electrodes (Tucker-Davis Technologies) both in vitro (saline, bottom traces) and in vivo (rodent cortex, top traces). The dashed lines indicate the impedance predicted thermal noise spectrum, the solid lines show measured noise, and shading indicates the variance. (**c**) Total integrated noise (TIN) for the representative silicon microelectrode array and (**d**) microwire array, in vitro (bottom traces) and in vivo (top traces), with measured noise (solid) and impedance predicted thermal noise (dashed). Colored shadows show variance within each electrode array. The predicted thermal noise is shown to accurately reflect the measured TIN at high frequencies, indicating thermal noise is the dominant noise source for wideband applications like rapidly multiplexed recording.

Recently, we performed wideband spectral measurements of implanted electrodes [37,38,39,40] (Figure 3). These measurements characterize the noise at high frequency as thermal in origin, on the basis of impedance magnitude and phase measurements. The measured high-frequency noise PSD is generally <20 nV_rms_/$\sqrt{\mathrm{Hz}}$ for typical penetrating microelectrodes. Background biological noise is generally in the 5–10 µV_rms_ range in the AP band [17], which would correspond to 75–150 nV_rms_/$\sqrt{\mathrm{Hz}}$ white noise across 0.5–5 kHz. Our studies [38] have also shown that high-frequency electromagnetic interference can be eliminated through proper referencing, grounding, and shielding, and that biological noise is confined to low frequencies (<10 kHz) [37]. Overall, thermal noise is the dominant concern at high frequencies and can be accurately predicted by impedance magnitude and phase measurements [38].

The thermal noise originates from the real part of the impedance within the bioelectrochemical cell, and can be predicted using

(9) vn2(f)=4kTRe[Z(f)],

where *v_n_*^2^(*f*) is the voltage noise power spectral density, *k* is Boltzmann’s constant, *T* is absolute temperature, and Re[*Z*(*f*)] is the real part of the impedance between the measurement point and ground [41]. This prediction is highly accurate for in vitro measurements, as well as in vivo for frequencies above typical biological bandwidths. Figure 3a,b shows in vitro and in vivo spectral measurements of a representative 16-channel tungsten microwire array (Tucker-Davis Technologies, Alachua, FL, USA) and a representative 16-channel silicon “Utah” microelectrode array [42] (Blackrock Microsystems, Salt Lake City, UT, USA), respectively. The in vivo measurements were performed under isoflurane anesthesia, which is known to suppress local cortical spiking activity [43], revealing the baseline noise floor. The real part of the measured impedance was used to predict the thermal noise (dashed lines), whereas the solid lines are direct spectral measurements [38]. For the in vitro measurements, the measured noise and predicted thermal noise match well across the spectrum. For in vivo measurements, there is excess low-frequency noise/activity attributed to biological sources other than local AP activity [37,38,39,44]. 

Figure 3c,d shows the running integral of the measured PSDs in vitro and in vivo for silicon and microwire arrays, respectively. The total integrated noise (TIN), which quantifies the noise floor of the system, is thermally dominated as shown by the accuracy of the predicted and measured TIN. The accuracy of the predicted thermal TIN indicates that the real part of the impedance can be used as an accurate estimator of high-frequency noise from the electrode‒tissue system.

**Figure 4:**
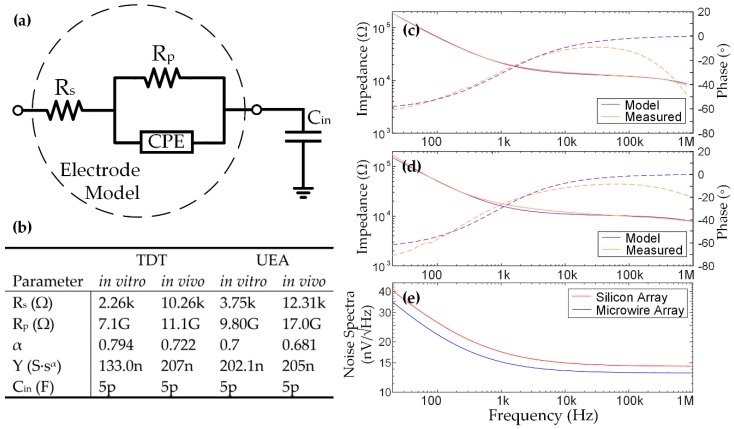
(**a**) Randles equivalent circuit electrode model. R_s_ represents the access resistance, which is the summation of several factors including electrode material, tissue encapsulation, protein binding, and cellular morphology between the recording and reference electrodes [47]. Faradaic reactions are represented by R_p_, which is also called the charge-transfer resistance and is representative of electrode surface oxidation and reduction reactions [27]. (**b**) Calculated model parameters in vitro and in vivo. (**c**) Measured and modeled in vivo impedance magnitude and phase for the representative silicon microelectrode array. (**d**) Measured and modeled in vivo impedance magnitude and phase for the representative microwire array. (**e**) Model predicted thermal noise neglecting the effect of C_in_.

Because the impedance is a good predictor of noise, it is useful to describe the electrode‒tissue interface with an equivalent circuit that can be used for simulation and design. The overall impedance is frequently modeled using a Randles equivalent circuit [45,46], often using a constant phase element (CPE) for accurate representation of both magnitude and phase:

(10) $$Z_{CPE}=\;\frac{1}{Y_{0}{\left(j\omega \right)}^{\alpha }},$$

where ω is radial frequency and $Y_{0}$ and α are fitting parameters. A simple model is shown in Figure 4 [27,47], where R_s_ is the access resistance, R_p_ represents faradaic reactions, the CPE models the interface’s double-layer capacitance, and C_in_ is the input capacitance of measurement instrumentation or acquisition electronics. C_in_ can interact with the high impedance of the electrodes to create a high-frequency pole, and is therefore important to the model matching. Parameters were extracted using the Gamry E-chem Analyst software (v7.06) for fitting the Figure 4a model to typical electrode impedance measurements (example parameters are shown in the figure). Figure 4c,d shows an example in vivo impedance and phase measurement compared to the fitted model for the microwire and silicon arrays, respectively. There is a high degree of agreement in impedance and low frequency phase. The high frequency phase shows a roll off incongruent with the input capacitance, but can be modeled with a capacitor parallel to the electrode model (not shown since the physical mechanism is unclear). The accuracy of the model in conjunction with the accuracy of the thermal noise prediction using Equation (9) indicates that an accurately parameterized model is a good method for predicting total thermal noise when designing neural recording electronics in general. This prediction is shown in Figure 4e for both types of arrays, neglecting C_in_ so that the prediction is system independent.

**Figure 5:**
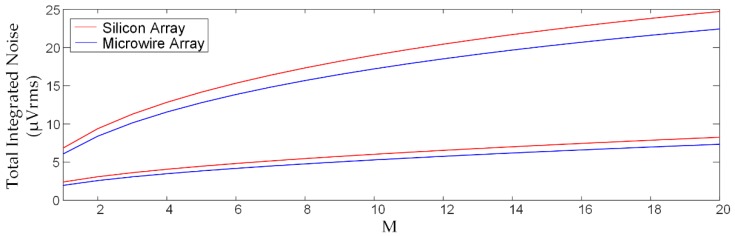
Comparison of voltage sampling (VS, top pair) and windowed integration sampling (WIS, bottom pair) for the representative silicon and microwire arrays using the Figure 4 model with a per channel sampling rate set to *f_ch_* = 30 kHz. Voltage sampling results in prohibitively large noise even for *M* = 4, whereas WIS allows for *M* = 20 with acceptable noise performance. A design should ideally account for the thermal noise along with expected biological noise and signal amplitudes in order to optimize for in vivo signal detection.

This model can be used to assess the impact of high-frequency noise on rapidly multiplexed recording. Figure 5 shows how the overall thermal TIN is affected by the multiplexing factor (M), with a comparison between voltage sampling and WIS (see Section 2.2). The curves were generated using Equations (3) and (7), and the model parameters in Figure 4. Figure 5 shows that WIS provides a roughly 3× decrease in the thermal TIN root-mean-square (rms) amplitude compared to voltage sampling. Since the dominant noise source at high frequencies is R_s_, the total integrated noise can be linearly extrapolated to higher frequencies to assess higher multiplexing factors. It can be seen that rapid multiplexing using voltage sampling becomes impractical for M values as low as 4 when budgeting for a <10 µV_rms_ thermal noise contribution, while WIS allows ~7 µV_rms_ for M = 20.

## 3. Rapidly Multiplexed Neural Recording Circuit Architecture

**Figure 6:**
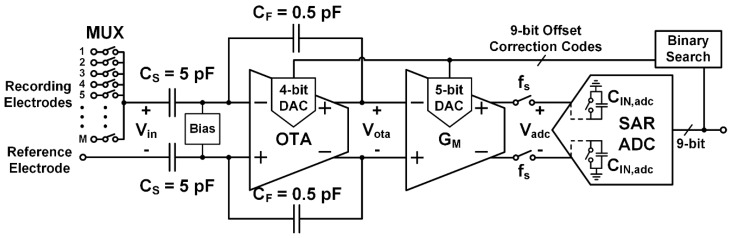
Diagram of the proposed rapidly multiplexed neural recording architecture. A capacitive feedback amplifier is used for pre-amplification with a gain of 10. Windowed integration sampling (WIS) is implemented with an open-loop transconductor ($G_{M}$) driving the input capacitance ($C_{\mathrm{IN},\mathrm{adc}}$) of a successive approximation ADC. Mixed-signal feedback is used to reject DC offsets between the recording and reference electrodes by injecting correction signals through digital-to-analog converters (DACs) in the analog signal path.

The architecture shown in Figure 6 was developed to address the acquisition issues discussed in Section 2. The first stage is a capacitive feedback low noise amplifier (LNA) based on an operational transconductance amplifier (OTA), which is followed by an open-loop transconductance amplifier ($G_{M}$) that forms a windowed integration sampler in combination with the input capacitance ($C_{\mathrm{IN},\mathrm{adc}}$) of a successive approximation (SAR) ADC. The prototype system supports up to 32 multiplexed electrodes (M= 1–32), and is designed to generate samples of each electrode at $f_{ch}=$ 30 kHz by rapidly multiplexing the electrodes at $\;f_{s}=M\cdot f_{ch}$, which is 300 kHz to 1 MHz depending on M.

When each electrode is selected, the DACs in the OTA and G_M_ amplifiers are updated to cancel DC offsets. The offset correction DAC codes are calculated using a binary search algorithm that processes the acquired data from the ADC. Since the electrode DC offsets do not change rapidly, the DAC codes can be recalculated every second (1 Hz). The LNA implements a closed-loop gain of 10 given by $C_{S}/C_{F}$, which relaxes noise requirements on the subsequent stages. Since the LNA gain is fairly low, it allows residual uncorrected offset from the electrodes and the OTA itself to pass without causing clipping at V_ota_ (Figure 6). The WIS operation provides additional voltage gain given by $G_{M}T_{s}/C_{IN,adc}$, which is designed at 100 with M=20 ($T_{s}=$ 1.5 µs). Thus, an overall 60 dB passband gain is used for amplifying APs. The transconductor ($G_{M}$) uses a 5-bit DAC to remove residual offset at the LNA output, in order to maximize the useful dynamic range of the circuit and relax the ADC resolution requirement. Integration of the signal current onto $C_{\mathrm{IN},\mathrm{adc}}$ reduces the high-frequency noise from the LNA and the electrodes ($NEB_{wis}≅$ 333 kHz for $T_{s}=$ 1.5 µs, M=20). This WIS operation attenuates high-frequency noise before it aliases as a result of forming a discrete time sample (see Equations (6) and (7) in Section 2.2).

The multiplexer itself (MUX in Figure 6) contributes thermal noise due to the on-resistance of the switches, but this noise can be made negligible without requiring large switch sizes (e.g., 12 × 0.18 µm^2^ switches were used in this design, corresponding to 160 Ω resistance and 0.94 µV_rms_ noise). With small switches, charge injection and clock feedthrough are not significant given the 5 pF C_S_ capacitance and typical electrode double layer capacitances (~1 nF). Simulation and measurements of the fabricated prototype confirmed that charge injection and clock feedthrough do not significantly affect offset, noise, or linearity in this design.

**Figure 7:**
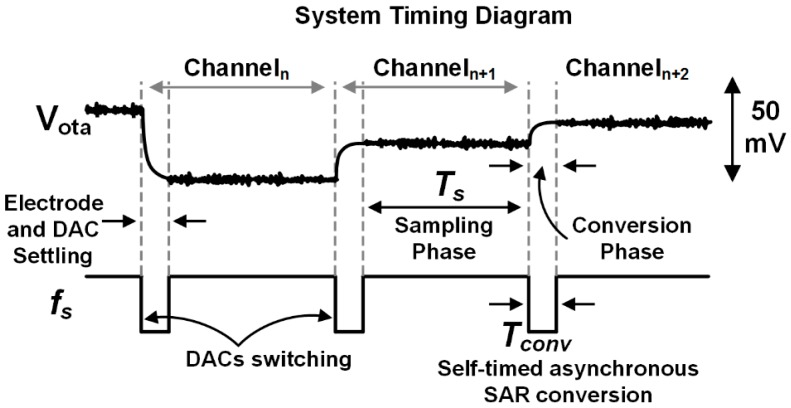
Illustration of the multiplexed recording circuit operation showing the OTA output (top) along with timing information. The OTA DAC provides coarse cancellation of the electrode DC offsets. After a short period of time ($T_{conv}$) reserved for settling of the electrode, LNA, and DACs, the electrode signal is integrated onto the input capacitance of the ADC ($C_{\mathrm{IN},\mathrm{adc}}$).

Figure 7 shows a timing diagram of the circuit operation, with an illustration of the LNA output (V_ota_). When an electrode is selected, a brief period of time ($T_{conv}$) is reserved to allow the electrode signal to settle through the LNA before the $G_{M}$ amplifier is connected and WIS begins. During the $T_{conv}$ phase the sample from the last electrode selected is also being digitized by the ADC, which uses an asynchronous, self-timed controller that does not require a clock [48]. $C_{\mathrm{IN},\mathrm{adc}}$ is reset subsequently before the $T_{s}$ phase begins. For the current prototype implementation, $T_{conv}$ > 110 ns is required for ADC conversion, allowing the WIS integration time ($T_{s}$) to be ≥930 ns for M=  1–32. In general, the NEB reduction from WIS depends on the timing overhead that $T_{conv}$ takes away from the maximum possible $T_{s}$. However, even at M = 32, $T_{conv}$ only takes up 11% of the total available period ($T_{conv}+T_{s}$) and hence does not severely degrade the NEB reduction (~3× versus voltage sampling). The LNA provides fast voltage settling, since the bias current required to reduce circuit noise results in a large bandwidth (7.5 MHz translating to $\varepsilon_{dtol}=$ 0.1% at the end of $T_{conv}$).

### 3.1. LNA Design

**Figure 8:**
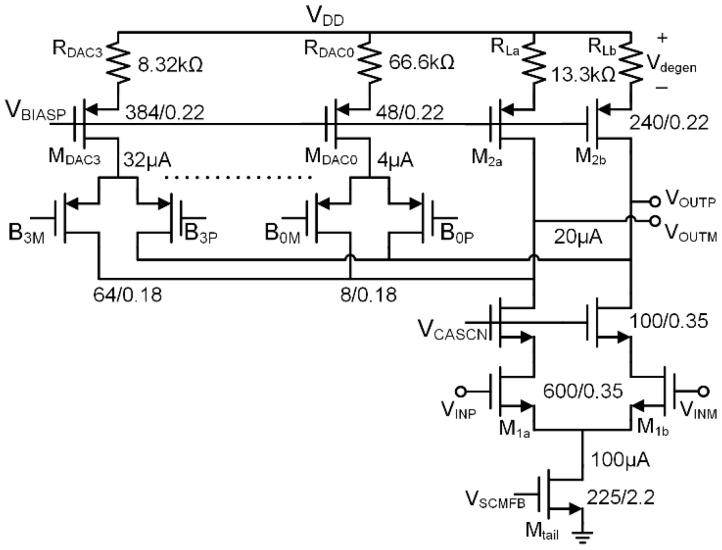
Transistor level design of the OTA in Figure 6. The topology leverages the low output signal swing requirements by using an efficient single stage structure. Coarse offset correction is achieved with a 4-bit digital-to-analog converter (DAC) implemented with binary weighted active load slices that steer current between the differential outputs.

A closed loop capacitive feedback topology was chosen for the LNA, since it provides good linearity, well controlled gain, and ease of input biasing. The transistor level implementation of the OTA amplifier, shown in Figure 8, is optimized for noise and power efficiency. The topology consists of a cascoded NMOS differential pair, and resistor-degenerated active loads split into parallel branches to implement a 4-bit offset correction DAC. The output voltage (V_ota_ in Figure 6 and Figure 7) does not experience high signal swing, because APs typically produce amplitudes <1 mV peak on extracellular electrodes, and electrode DC offsets are suppressed by the LNA’s offset correction DAC (+/−5 mV residual input referred offset). Thus, an efficient single stage OTA topology can be used, which is power efficient and easy to stabilize (load compensated).

The differential pair transistors (M_1a,b_) operate in weak inversion and were sized in order to optimize the tradeoff between thermal noise and flicker noise. This tradeoff is essentially the same as with traditional capacitive feedback amplifiers used in neural recording (see Section 2.2), and has been studied extensively [17]. The degenerated active loads have a *g_m_R* product of 6, to suppress flicker noise from the PMOS devices as much as possible given the available headroom (V_degen_ ≈ 260 mV). The LNA was designed for an input referred noise of 5 µV_rms_ within a NEB of 333 kHz (M=20), which translates to a 8.7 nV/$\sqrt{\mathrm{Hz}}$ spectral density. Therefore, each branch of the differential pair was biased at a drain current of 50 µA, resulting in a unity gain bandwidth of ~7.5 MHz, which is sufficient for 0.1% settling in 166 ns ($T_{conv}$). Since electrode offsets imbalance the differential pair, the noise of M_tail_ is not entirely canceled. Therefore, its *g_m_*/*I_D_* was made relatively low (16 V^−1^).

A 4-bit current source DAC topology was chosen here for offset correction, which allows high switching speed (~166 ns settling in this design). One can derive a relation between the input offset and the compensating current imbalance that the DAC must inject:

(11) $$V_{os,in}=V_{INP}-V_{INM}=\frac{C_{F}+C_{S}+C_{P}}{C_{S}}nU_{T}\ln\left(I_{D1b}/I_{D1a}\right),$$

where $V_{INP,M}$ are the input voltages of the OTA, $C_{F}$ and $C_{S}$ are the feedback network capacitances shown in Figure 6, $C_{P}$ is the parasitic input capacitance of the OTA, n is subthreshold slope factor, $U_{T}$ is the thermal voltage, and $I_{D1a}$ and $I_{D1b}$ are the drain currents in the differential pair. The 4-bit offset correction DAC can compensate ±65 mV of electrode offset, using a least significant bit (LSB) size of 4 µA that translates to 10 mV input referred offset. This DAC can also correct for the offset of the amplifier itself, although the OTA’s offset is far below the LSB size (σ = 400 µV input referred based on Monte Carlo simulation). Residual electrode offset and the OTA’s offset are corrected by the fine correction DAC in the G_M_ amplifier, described below. The low gain of the LNA (10) ensures that residual offset does not saturate its output, and the fine correction DAC in the G_M_ amplifier ensures that the final signal chain output is not saturated by offset despite the 60 dB overall passband gain.

The input node biasing circuit of the LNA (Bias in Figure 6) consists of reset switches, which periodically (>1 s) connect the input nodes to a reference voltage. The OTA also uses a switched capacitor common mode feedback circuit (not shown) connected to M_tail_, which is split with a 20% fixed bias segment to facilitate startup.

### 3.2. Transconductance Amplifier Design

**Figure 9:**
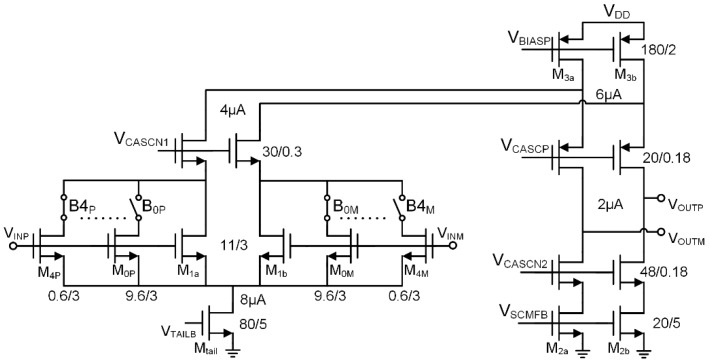
Transistor level design of the transconductance amplifier used in the windowed integration sampler (G_M_ in Figure 6). A folded cascode architecture was selected for high output resistance and moderate output swing. A 5-bit fine offset correction digital-to-analog converter was implemented through an array of differential pair devices.

The output of the transconductance amplifier (G_M_ in Figure 6) swings across the full scale range of the ADC (+/−0.9 V differential). A folded cascode topology (Figure 9) was chosen to satisfy this swing requirement while achieving high output resistance to avoid gain error in the WIS operation [34]. Given the relaxed 1/*f* noise requirements provided by the LNA’s gain, the input differential pair devices (M_1a,b_ in Figure 9) do not need to be sized large. This led to the choice of implementing the 5-bit fine offset correction DAC by changing the size of the input pair [28], resulting in low area overhead and fast settling (<166 ns). An alternative would be a capacitive DAC at the G_M_ input nodes [18], but this approach would require capacitive coupling between the LNA and G_M_ amplifiers, and the input pair DAC is a simpler implementation.

For an approximate understanding, the weak inversion current equation can be used to derive the offset referred to the G_M_ input nodes, which leads to [28]:

(12) $$V_{os}=nU_{T}\ln\left(W_{1a}/W_{1b}\right),$$

where $W_{1a,b}$ are the total widths of the input pair devices, which change as a function of the DAC codes. The LNA passes a worst case residual offset of approximately 50 mV at its output. The G_M_ DAC’s offset correction range was designed for ±57 mV to leave some margin for mismatch effects and for ease of DAC sizing. 

A switched capacitor common mode feedback circuit (not shown) was connected to the output active load transistors (M_2a,b_), which were split with a 25% fixed bias segment to facilitate startup.

### 3.3. SAR ADC Design

A successive approximation register (SAR) ADC was chosen for low power dissipation, adequate conversion speed, and for streamlined integration with the WIS operation. Moderate resolution (8–10 bits) is acceptable given the offset correction DACs, which preserve useful dynamic range for AP signals. Small CDAC unit capacitors (2.4 fF) [49] and asynchronous digital control [48] were used to reduce power and area in the ADC. The 9-bit design uses top-plate sampling on an 8-bit CDAC, with a monotonic switching procedure [48]. A fully dynamic latch based comparator was used for low power consumption. The CDAC consists of split capacitor elements to maintain a constant common mode voltage at the comparator inputs [50]. The total CDAC capacitance is approximately 600 fF ($C_{\mathrm{IN},\mathrm{adc}}$ in Figure 6), and is constructed with custom metal-oxide-metal (MOM) capacitors in three metal layers. The asynchronous, self-timed SAR controller simplifies clocking by only requiring a master sample clock, and can achieve sample rates up to 9 MHz in this design.

## 4. Experimental Results

### 4.1. Bench Testing of the CMOS Prototype

**Figure 10:**
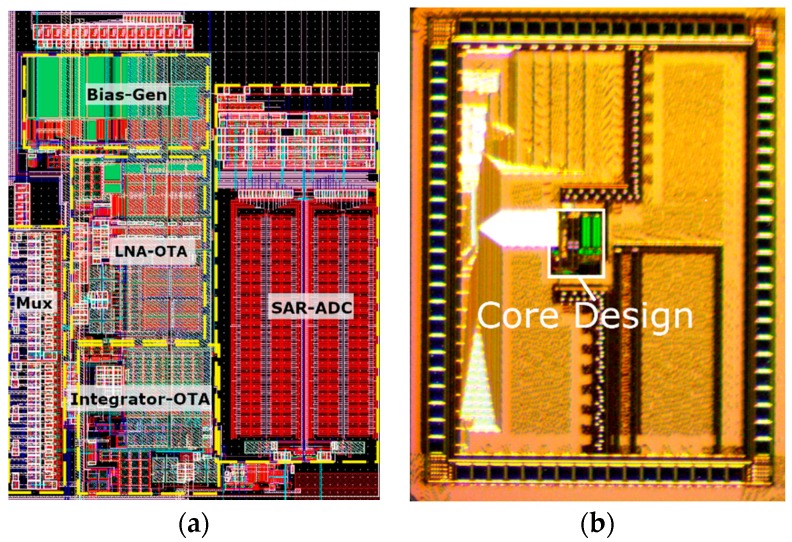
(**a**) Layout of the core circuitry of the fabricated CMOS design; (**b**) micrograph of the fabricated test chip, with core circuitry highlighted in the center.

The rapidly multiplexed circuit described in Section 3 was implemented in the ON Semiconductor 180 nm CMOS process with 6 metal layers. The micrograph of the fabricated test chip is shown in Figure 10. The chip contains a 32:1 input multiplexer, the core circuits described in Section 3, and programmable bias generator blocks. A master clock is generated externally and used for multiplexer control, DAC updating, and common mode feedback circuit clocking. Reference voltages for the LNA input bias, common-mode feedback circuits, and ADC were generated off-chip. The design has a core circuit area of 245 µm × 315 µm, while the overall test chip itself is pad-limited and is 1.7 mm × 2.3 mm. The chip was mostly tested for a multiplexing factor of M= 20, with a multiplexing clock rate of 600 kHz. The power supply voltage is 1 V and the total power consumption is 140 µW, which translates to 7 µW per channel for M= 20. The test chip was packaged in an 80 pin, 12 mm × 12 mm thin quad frame package (TQFP). Test PCBs consist of a motherboard for various functions and connections, and a daughterboard to hold the test chip.

The digital and analog inputs to the chip as well as the ADC outputs were all processed through a National Instruments (NI; Austin, TX, USA) platform consisting of a multifunction data acquisition card (DAQ, PXIe-6368) and a high-performance arbitrary waveform generator (AWG, PXIe-5451). The test system was controlled with the NI DAQmx API through Python 2.7. The binary search algorithm for finding offset correction codes (Figure 6) was also implemented in Python for flexibility when prototyping. Data processing was performed in Python and MATLAB.

Gain was measured using an attenuated sinusoidal input from the AWG, and reading of the ADC output codes. Gain was measured at 59.1 dB, and is flat up to the 15 kHz channel Nyquist frequency. Bandwidth limiting for AP detection is accomplished with software digital filters (see Section 4.2 below). The total harmonic distortion (THD) was measured using an input signal from the AWG and a fast Fourier transform (FFT) of the ADC output codes. Simulations showed a worst case THD at the LNA output to be 1.5%, while measurements of the full chain THD (including integrator and ADC) indicated 2%. The common mode rejection ratio (CMRR) was simulated at the LNA output with an input offset of 63 mV and the maximum offset correction DAC settings, indicating a worst case CMRR of 65 dB. The measured worst case CMRR was 50 dB for the full chain, which is likely due to mismatch in the circuitry.

**Figure 11:**
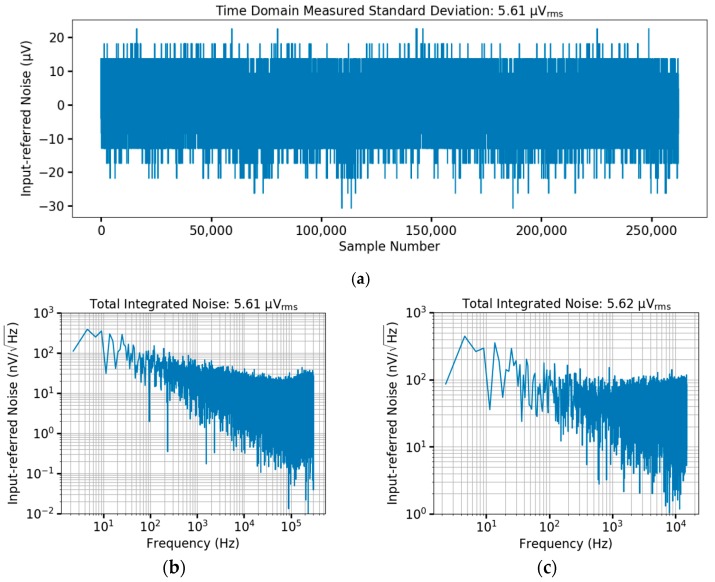
Full system input-referred noise measured from the ADC output codes. (**a**) Time domain plot of 262,144 ADC samples (400 ms time record); (**b**) Single channel FFT spectrum with sampling frequency of 600 kHz; (**c**) Spectrum of demultiplexed samples with 30 kHz sampling per channel.

**Figure 12:**
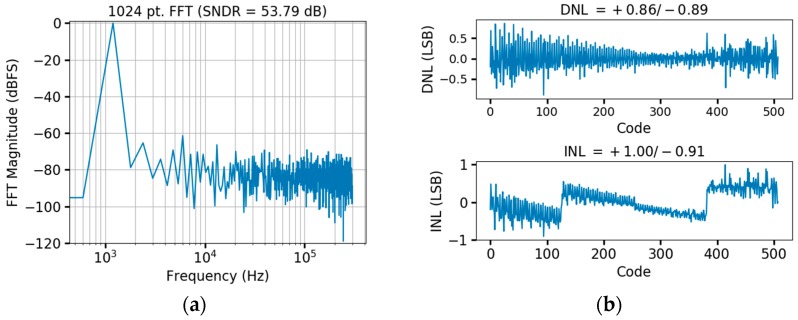
ADC test results. (**a**) FFT with 1 kHz input sinusoid showing 53.8 dB SNDR; (**b**) Linearity measurements showing DNL of +0.86/−0.89 and INL of +1.00/−0.91.

The input referred noise of the overall circuitry was measured from the ADC output codes with a grounded input (Figure 11), resulting in 5.6 µV_rms_. With a total chip current of 140 µA, bandwidth of 15 kHz (set by the ADC Nyquist frequency), and M= 20, the noise efficiency factor (NEF) [51] is 4.74 when considered on a per channel basis, which is within the range of traditional neural recording circuitry [17]. The ADC was measured by itself for signal to noise plus distortion ratio (SNDR) with a 1 kHz sinusoidal input delivered through auxiliary test pads, resulting in 53.8 dB (ENOB of 8.3 bits) as shown in Figure 12a. The power consumption of the ADC is 6.5 µW at 600 kHz (325 nW per channel for M= 20), corresponding to 37 fJ per conversion step. The DNL and INL were measured using the histogram method, indicating a DNL of 0.86/−0.89 and INL of 1/−0.91 as shown in Figure 12b. Parasitic extraction simulations of the OTA demonstrate a gain bandwidth product (GBW) of 7.84 MHz and phase margin of 90°, compared to 7.67 MHz and 90° from nominal simulations. The higher GBW after layout extraction is due to differences in device fingering. Monte Carlo simulations indicate σ = 400 µV input referred offset for the OTA (200 runs), which is consistent with measurement results across three chips. Parasitic extraction of the overall test chip indicates 270 fF of capacitive loading from wiring, pads, ESD, and device parasitics, while the 5 pF C_S_ capacitance of the LNA presents 6.7 MΩ impedance when periodically connected to a given electrode at $f_{ch}$ = 30 kHz. This input loading is commensurate with traditional neural recording circuitry [17], and can be reduced if needed for particular microelectrodes through feedback strategies typically used in chopper amplifiers [52,53].

Table 1 shows the power and area of the individual circuit blocks shown in Figure 6 and Figure 10a. The power is dominated by the LNA, since it dominates the input referred noise of the design. The area contributions of the LNA, G_M_ block, and the ADC are similar, with the ADC being the largest because of its CDAC. It should be noted that the ADC area in particular would be reduced greatly if implemented in a smaller CMOS process node. Table 2 summarizes the bench measurements of the design for M= 20 and compares the performance to state-of-the-art traditional AP recording circuits. This design achieves the lowest area per channel for AP recording, while being competitive in the rest of the specifications. This was achieved in an older CMOS process node and with a conventional capacitive feedback LNA design, demonstrating the efficacy of the rapidly multiplexed approach. Of particular note is that the power per channel and NEF per channel metrics are in the same range as traditional neural recording circuits, demonstrating that there is no fundamental advantage or disadvantage to the approach in terms of power dissipation. For offset correction, this work used dynamic offset correction DACs, similarly to the single channel design presented in [28]. The work in [54] pursues analog-to-time conversion, with extensive use of digital blocks that benefit from CMOS process scaling. However, that work needs to be extended to a multi-channel architecture and should deal with electrode offsets that are modulated by the chopping technique. Finally, this work achieves over an order of magnitude reduction in the area per channel compared to state-of-the art multichannel designs [55,56,57]. The technique of rapidly multiplexed AP acquisition is unique to this work, and the proof of concept demonstration should be viewed as the starting point for further circuit innovation and optimization.

### 4.2. In Vivo Testing of the CMOS Prototype

One 16-channel (2 × 8) tungsten microwire array (Tucker-Davis Technologies) was implanted into the cortex of a male Sprague Dawley rat (500 g). The array was customized to have varying shaft lengths from 1 to 3.8 mm in length, and was positioned perpendicular to the midline to allow for recording from the more lateral barrel cortex as well as the motor cortex. All studies were conducted with the approval of the Institutional Animal Care and Use Committee at the University of Utah. The surgical procedure was similar to that outlined in [38]. Anesthesia was induced using 5% vaporized isoflurane in a specialized induction chamber and maintained at 1.5–3%. Two incisions (approximately 2.5 mm apart) were made along the midline of the skull. Two more incisions were made to connect the tops and bottoms and create a rectangular opening. Blunt dissection was used to separate the skin from the underlying fascia. Four bone screws were inserted into the skull: one in each corner of the exposed skull surface along the medial face of the temporal ridge. An approximately 3.5 mm diameter craniotomy was performed over the insertion site using a hand drill. The underlying dura was then incised and manipulated using a 26 G needle to expose the cortex. The array was slowly inserted into the tissue using a stereotaxic arm with the longer 3.8-mm shanks most lateral and the 1-mm shanks most medial from the midline. After the desired depth was reached, the craniotomy was filled with Kwik-Cast silicone elastomer (World Precision Instruments, Sarasota, FL, USA) and the skull was covered with UV cure epoxy to protect and stabilize the array.

All recordings were performed in the same experimental session (two days after surgery) on a single rat implanted with one microwire array as described above. During the recording session, the animal was anesthetized using a ketamine (70 mg/kg)/xylazine (10 mg/kg) cocktail. To better assess the multiplexed measurements, additional recordings were made using the well-established Cerebus Neural Recording system (v.6.04.02, Blackrock Microsystems). Each of the 16 recording channels used in the Cerebus system produced a dual output: a continuous time data stream with an analog bandpass filter from 0.3 Hz to 7.5 kHz, and a second channel with a digital filter from 750 Hz to 7.5 kHz used for action potential (AP) detection. Both channels used a 30 kHz sampling frequency.

**Figure 13:**
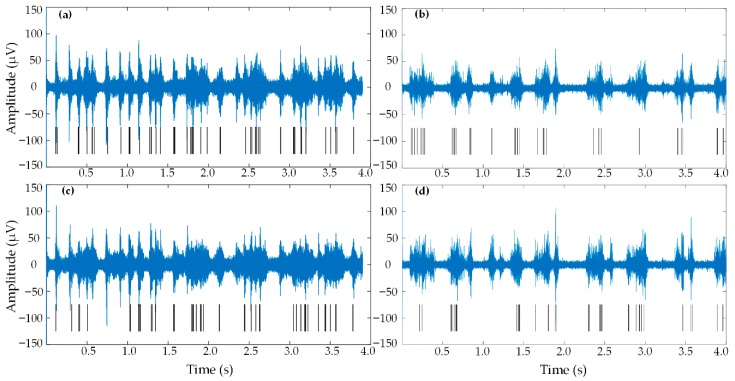
Continuous data from the rapidly multiplexed CMOS test chip (left column) and the Cerebus system (right column). All data were filtered from 800 Hz to 4 kHz using a digital bandpass filter implemented in MATLAB. Black lines under each plot indicate threshold crossing events. The top row contains data from the same electrode recorded from the test chip (**a**) and Cerebus (**b**). The second row contains data from a second electrode recorded from the test chip (**c**) and Cerebus (**d**).

**Figure 14:**
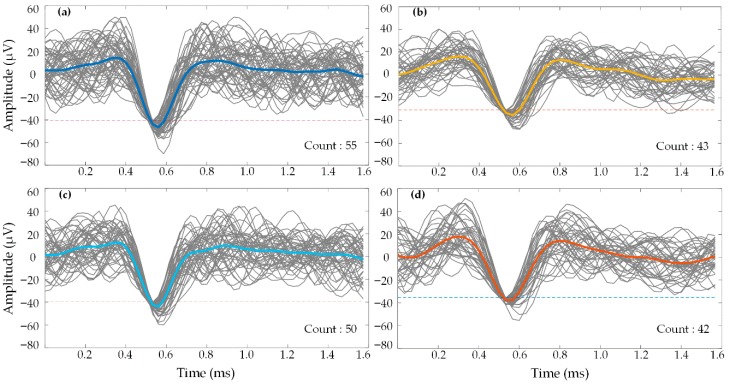
Waveforms of threshold crossing events recorded from the rapidly multiplexed chip (left column) and Cerebus (right column) extracted from the data shown in Figure 14. (**a**,**b**) show waveforms from one electrode, (**c**,**d**) are taken from a second electrode. Bold, colored traces represent the mean of each electrode’s threshold crossing waveforms. Dotted lines indicate the threshold.

Figure 13 shows continuous recordings from two different electrodes selected for their robust threshold crossing activity, each recorded from the rapidly multiplexed CMOS test chip and the Cerebus system (not simultaneously). In Figure 13a,c, data are shown from the two electrodes when multiplexed and sampled at 600 kHz, corresponding to an effective multiplexing factor of M = 16. The 300 kHz data streams were then demultiplexed and downsampled to 37.5 kHz by throwing away samples, using custom MATLAB software. The same two electrodes recorded from the Cerebus system were sampled from two different channels at a rate of 30 kHz, and data are shown in Figure 13b,d. All four traces show periodic bouts of high-amplitude bursts strongly correlated with threshold crossing events. Figure 14 shows the preservation of similar wave shapes and peak-to-peak amplitudes across the two recording systems. All events come from the same 4-s recordings shown in Figure 13, which displays the Figure 14 threshold crossing events with the raster plot at the bottom of each panel. Spike sorting was performed using custom MATLAB software with time-amplitude window thresholds similar to the “hoops” described in [58]. Some variations in the averaged threshold crossing waveforms appear as patterns in the pre- and post-crossing segments. These minor patterns result from averaging a limited number of threshold crossing events (the number of events is shown in each panel of Figure 14), and are similar between the two recording systems.

The signal-to-noise-ratio was calculated as the peak-to-peak amplitude of the mean waveform divided by the standard deviation of noise in the waveform [59]:

(13) $$SNR=\;\frac{max\left(\bar{W}\right)-\min\left(\bar{W}\right)}{SD_{\varepsilon }},$$

where $\bar{W}$ is the mean waveform and ε is a matrix containing the difference of each point of individual waveforms from the mean. The two electrodes recorded with the test chip (Figure 13a,b) were found to have SNRs of 2.1 and 2.2, respectively. The same two electrodes when recorded from with the Cerebus system had SNRs of 2.4 and 2.2, respectively, showing good agreement between the two recording systems. The similarity of the acquired data between the CMOS test chip and the Cerebus system provides confidence in the ability to acquire APs using the rapidly multiplexed approach. The test chip results in a slightly higher background noise level of 9.9 µV_rms_ for Figure 13a and 10.1 µV_rms_ for Figure 13c, compared to the Cerebus recordings corresponding to 6.6 µV_rms_ for Figure 13a and 7.8 µV_rms_ for Figure 13b (computed by removing threshold crossing events from the overall waveform). This modest increase in the background noise is expected given the additional circuit noise and electrode thermal noise (5.6 µV_rms_ and roughly 6 µV_rms_ across the 18.75 kHz Nyquist zone, respectively).

## 5. Discussion

This report describes a new approach to acquiring neuronal action potentials from multichannel electrode arrays, based on time domain multiplexing of multiple electrode sites to a single integrated circuit. The implications of electrode DC offsets and high-frequency noise were discussed. Windowed integrator sampling was presented as an approach to mitigate high-frequency noise from the electrodes as well as electronics, enabling far higher multiplexing ratios than traditional voltage sampling. A CMOS integrated circuit architecture was proposed, which incorporates the windowed integrator sampling technique as well as mixed-signal DC offset cancellation. Transistor level design details of a proof-of-concept implementation were also presented. Finally, experimental results were reported from bench testing of the CMOS circuitry as well as acquisition of putative action potentials (possibly multi-unit) from a standard microwire electrode array implanted in rodent cortex.

The proposed approach replaces traditional multichannel neural recording circuitry with a single circuit that acquires signals from multiple electrodes. Sophisticated circuit techniques were required to maintain noise and power performance at levels that are commensurate with traditional neural recording circuitry, while achieving a dramatic reduction in circuit area. This approach can be viewed as combining the power dissipation of many traditional neural recording channels into a single circuit with higher bandwidth, leveraging high-speed operation. The reduction in circuit area, and the potential for further reduction, is critical for scaling neural recording systems to higher channel counts by enabling fully implantable electronics that are better matched to the size and density of emerging electrode arrays technologies [13,14,15]. Limitations on the multiplexing ratio imposed by high-frequency electrode noise still dictate that a number of copies of the circuitry be used to support electrode arrays with hundreds of channels and beyond, but the approach is compelling in terms of reduced area per channel as well as reduced complexity at the system level.

The rapidly multiplexed acquisition approach is particularly well suited for high channel count microelectrode arrays, where active circuitry is integrated with the device through homogenous fabrication [15,19,25] or advanced heterogeneous approaches [29,60,61]. The technique in itself does not address interconnect limitations that arise when adopting headstage recording architectures where active circuits (chips) are connected to electrode arrays through standard printed circuit boards and connectors (i.e., not fully implantable) [3,4,5]. Standard CMOS I/O bondpad dimensions often result in “pad-limited” implementations, such as the test chip shown in Figure 10b. However, the rapidly multiplexed approach does improve those architectures as well, since it allows for more chip area that can be used for signal processing, data compression, and communication circuits, which are increasingly important. The main goal of this report is to provide proof-of-concept evidence that rapid multiplexing, directly at the electrodes, without preamplification, is feasible for acquiring action potentials from multichannel electrode arrays. Future work should address fully implantable integration with arrays, and assessment of long term reliability. Issues must be investigated such as thermal considerations, dissolution of electrode materials, stability of packaging and encapsulation, and long term tissue response. These considerations are critical for any fully implantable active array, and rapid multiplexing does not fundamentally present new barriers (e.g., the power dissipation and electrode loading are commensurate with traditional neural recording circuitry [17]). Nevertheless, thorough studies of electrode behavior in the context of rapid multiplexing should be explored, and is part of our own ongoing work. In vivo characterization of a wider range of electrode array technologies should also be pursued, with rapid multiplexing in mind.

It is expected that many further improvements can be made. This report is intended to highlight new avenues of research in integrated circuits, microelectrode array design, and signal processing methods. Co-design of rapidly multiplexed systems across these three dimensions is a particularly interesting goal. Directions for future work in CMOS circuit design include offset cancellation techniques as well as noise mitigation techniques. Windowed integrator sampling and mixed-signal feedback were shown to be effective approaches, but there are likely others as well. There is also significant room for improvement in the core amplifier and ADC circuits beyond the prototype presented in this work, e.g., leveraging more advanced IC process technologies.

The results indicate that rapidly multiplexed action potential acquisition without preamplification is possible, which to our best knowledge has not been shown before. Overall, this report demonstrates a compelling candidate approach for scaling up neural recoding systems.

## Figures and Tables

**Table 1:** Area and power of distribution among design blocks.

Block	Power	Area
MUX	0.6 µW	0.0059 mm^2^
LNA	110 µW	0.011 mm^2^
G_M_	12 µW	0.009 mm^2^
SAR ADC	6.5 µW	0.025 mm^2^
Bias-Gen	10 µW	0.0087 mm^2^

**Table 2:** Measured performance of the rapidly multiplexed CMOS design (M= 20) and comparison to state-of-the-art neural recording circuits.

Parameter	[28]	[54]	[55]	[56]	[57]	This Work
Process	65 nm	65 nm	180 nm	180 nm	65 nm	180 nm
Supply Voltage	0.5 V	0.5 V	0.45 V	0.5**–**1.8 V	1 V	1 V
Supply Current per Channel	10.08 µA	2.55 µA	1.6 µA	18 µA	3.28 µA	7 µA (140 µA total)
Gain [V/V]	N/A	N/A	52	N/A	52.1	59.1
Bandwidth	10 kHz	11 kHz	10 kHz	9.2 kHz	8.2 kHz	15 kHz
Input-Referred Noise [µV_rms_]	4.9	3.8	3.2	3.37	4.13	5.6
Noise Efficiency Factor	5.99	2.2	1.57	2.61	3.19	4.74
THD	2%	0.1%	N/A	N/A	1%	2%
CMRR	75 dB	60 dB	73 dB	60 dB	80 dB	50 dB
Circuit Area per Channel [mm^2^]	0.013	0.006	N/A	0.098	0.042	0.0039 (0.077 total)
